# Supplementary material for: Bifidobacterium longum BORI inhibits pain behavior and chondrocyte death, and attenuates osteoarthritis progression
Source: PLoS One. 2023 Jun 23;18(6):e0286456. doi: 10.1371/journal.pone.0286456 (PMC10289443; doi:10.1371/journal.pone.0286456)
Supplement: Attachment — Submitted filename: Rebuttal leter_BORI.docx [file pone.0286456.s001.docx]

Journal requirements:

When submitting your revision, we need you to address these additional requirements.

1. Please ensure that your manuscript meets PLOS ONE's style requirements, including those for file naming. The PLOS ONE style templates can be found at

<https://journals.plos.org/plosone/s/file?id=wjVg/PLOSOne_formatting_sample_main_body.pdf> and

<https://journals.plos.org/plosone/s/file?id=ba62/PLOSOne_formatting_sample_title_authors_affiliations.pdf>

**Resposne : We edited to match the PLOS ONE's style requirements of this manuscript.**

2. Thank you for stating the following financial disclosure:

“This research was supported by a grant of the Food Industry Promotional Agency of Korea and this research was supported by a grant of the Korea Health Technology R&D Project through the Korea Health Industry Development Institute (KHIDI), funded by the Ministry of Health & Welfare, Republic of Korea (grant number HI20C1496)”

Please state what role the funders took in the study.  If the funders had no role, please state: "The funders had no role in study design, data collection and analysis, decision to publish, or preparation of the manuscript."

If this statement is not correct you must amend it as needed.

Please include this amended Role of Funder statement in your cover letter; we will change the online submission form on your behalf.

**Resposne : "The funders had no role in study design, data collection and analysis, decision to publish, or preparation of the manuscript."**

3. Thank you for stating the following in your Competing Interests section:

“No”

Please complete your Competing Interests on the online submission form to state any Competing Interests. If you have no competing interests, please state "The authors have declared that no competing interests exist.", as detailed online in our guide for authors at <http://journals.plos.org/plosone/s/submit-now>

 This information should be included in your cover letter; we will change the online submission form on your behalf.

**Resposne : "The authors have declared that no competing interests exist."**

4. In your Data Availability statement, you have not specified where the minimal data set underlying the results described in your manuscript can be found. PLOS defines a study's minimal data set as the underlying data used to reach the conclusions drawn in the manuscript and any additional data required to replicate the reported study findings in their entirety. All PLOS journals require that the minimal data set be made fully available. For more information about our data policy, please see <http://journals.plos.org/plosone/s/data-availability.>

Upon re-submitting your revised manuscript, please upload your study’s minimal underlying data set as either Supporting Information files or to a stable, public repository and include the relevant URLs, DOIs, or accession numbers within your revised cover letter. For a list of acceptable repositories, please see <http://journals.plos.org/plosone/s/data-availability#loc-recommended-repositories.> Any potentially identifying patient information must be fully anonymized.

Important: If there are ethical or legal restrictions to sharing your data publicly, please explain these restrictions in detail. Please see our guidelines for more information on what we consider unacceptable restrictions to publicly sharing data: <http://journals.plos.org/plosone/s/data-availability#loc-unacceptable-data-access-restrictions.> Note that it is not acceptable for the authors to be the sole named individuals responsible for ensuring data access.

We will update your Data Availability statement to reflect the information you provide in your cover letter.

**Resposne : Thank you for your comment. We have updated the Figshare site.**

**10.6084/m9.figshare.22566634**

Reviewer comments
This paper submitted by the authors investigated whether B. longum BORI modulates arthritic symptoms and accompanying pain symptoms in an osteoarthritis model. Probiotics such as B. longum are currently being investigated and published for their disease-modulating effects in various inflammatory diseases. The results presented in this paper will add to our knowledge of probiotic therapies that can inhibit articular cartilage damage and pain in osteoarthritis models. I have several questions regarding this study.

1) In Background section authors present the topic well and with appropriate reference however little is said about the rational of their study. My suggestion is to expand on the relevance of in vitro models used, the bacteria strains they used, why they used that strains and the relevance these strains might have.

**Response:** We agree with referee’s comment. We revised the introduction section (marker by red color)

Monosodium iodoacetate (MIA)-induced OA rats are widely used as a human osteoarthritis model, by destroying cartilage cells, causing degeneration and inflammation of the cartilage[14]. This causes symptoms similar to those of thoracic osteoarthritis. The administration of *B.* BORI is safe and has demonstrated positive effects in terms of inhibiting inflammation in cranial nerve cells, as well as hepatic and intestinal conditions [10, 15]. Furthermore, B. BORI demonstrated the maintenance of gut microbiota balance and stability by showing effectiveness in preventing and treating rotavirus infection in infants[16].

2) The author seems to have partially mentioned the number of experiments in the experiment method and results. It is desirable that the total number of experiments performed in this part be accurately reflected.

**Response:** Thank you for your comments. Osteoarthritis was induced in Wistar rat (n=6). The experiment was performed two times. We revised the Material and method section (marker by red color)

3) Why are some experiments used after killing B. longum BORI? An explanation is needed as to why dead bacteria were used.

**Response:** Heat-killed probiotics are reportedly safer than live probiotics for eliminating antibiotic-resistance genes, preventing the production of recombinant strains, and controlling the microbial load during probiotic supplementation (Ishibashi, N. & Yamazaki, S. Probiotics and safety. Am. J. Clin. Nutr. 73,465S–470S (2001); Vintini, E.O. & Medina, M.S. Host immunity in the protective response to nasal immunization with a pneumococcal antigen associated with live and heat-killed *Lactobacillus* *casei*. BMC Immunol. 12, 46 (2011)). Thus, we elected to use heat‑killed *B. longum* BORI in this study.

4) In methods, "Preparation of bacteria" it is not mentioned what sample was used for bacteria isolation

The authors would like to thank the comment of the reviewer. Following the reviewer’s comment, the authors revised the reference as follows: (marker by red color)

B. longum BORI was isolated from human feces of healthy infant who lived in Chuncheon, South Korea between 1995 and 1998, identified with 16S rRNA sequencing and cultured with MRS (De Man, Rogosa and Sharpe, Becton Dickson, Franklin Lakes, NJ, USA) broth medium at 37 ◦C. For the initial screening, lyophilized powder of each strain was used. Freeze-dried powder of B. longum BORI was provided by BIFIDO Co. Ltd (Hongchun, Korea)

5)In the same section, "Immunohistochemical staining of joint synovium showed that administration of B. longum BORI reduced the levels of IL-1b, LTB4r, PGE2, MMP9 and MMP13 (Figures 3 and 4)." . Histology in the method of Figures 3, 4 and 4 is not clear how to observe the reduction of the listed cytokines. It is necessary to add a description of how the cytokine reduction is observed.

**Response:** We agree and added the following text in method section. (marker by red color)

The all IHC images were obtained each rat, and showing representative images. The percentage of positive cells showing in each image was measured by Image J.

6) The title refers only to experiments with rats. The experiment also includes the contents of the experiment in the patient's chondrocyte culture, so it would be good to mention these parts in the title.
**Response:** Thank you for your comment. We have revised the title to “

***Bifidobacterium longum* BORI reduces pain behavior and attenuates the progression of monosodium iodoacetate-induced osteoarthritis and**

🡪 ***Bifidobacterium longum* BORI inhibits Pain behavior and chondrocyte death, and attenuates osteoarthritis progression.**

7) In figure, B. longum BORI was not mentioned in Italics. It should be corrected throughout the Figure.
**Response:** Thank you for your comment. We revised the manuscript.

8) The authors should better check the manuscripts for any typographical errors.
For example, LTB4 must be modified to LTB4r in figure 3.

**Response:** We apologize for this error, which has been corrected.
